# Nicotinamide riboside and caffeine partially restore diminished NAD availability but not altered energy metabolism in Alzheimer's disease

**DOI:** 10.1111/acel.13658

**Published:** 2022-06-21

**Authors:** Woo‐In Ryu, Minqi Shen, Yoon Lee, Ryan A. Healy, Mariana K. Bormann, Bruce M. Cohen, Kai‐Christian Sonntag

**Affiliations:** ^1^ Department of Psychiatry, McLean Hospital Harvard Medical School Belmont Massachusetts USA; ^2^ Basic Neuroscience Division, McLean Hospital Harvard Medical School Belmont Massachusetts USA; ^3^ Program for Neuropsychiatric Research, McLean Hospital Harvard Medical School Belmont Massachusetts USA

**Keywords:** aging, Alzheimer's disease, bioenergetics, caffeine, induced pluripotent stem cells (iPSC), NAD, nicotinamide riboside

## Abstract

The redox co‐factor nicotinamide adenine dinucleotide (NAD) declines with age, and NAD deficits are specifically associated with dysfunctional energy metabolism in late‐onset Alzheimer's disease (LOAD). Nicotinamide riboside (NR), a dietary NAD precursor, has been suggested to ameliorate the aging process or neurodegeneration. We assessed whether NR with or without caffeine, which increases nicotinamide mononucleotide transferase subtype 2 (NMNAT2), an essential enzyme in NAD production, modulates bioenergetic functions in LOAD. In LOAD patients—and young or old control individuals—derived dermal fibroblasts as well as in induced pluripotent stem cell‐differentiated neural progenitors and astrocytes, NR and caffeine cell type‐specifically increased the NAD pool, transiently enhanced mitochondrial respiration or glycolysis and altered the expression of genes in the NAD synthesis or consumption pathways. However, continued treatment led to reversed bioenergetic effects. Importantly, NR and caffeine did not alter the characteristics of a previously documented inherent LOAD‐associated bioenergetic phenotype. Thus, although NR and caffeine can partially restore diminished NAD availability, increasing NAD alone may not be sufficient to boost or restore energy metabolism in brain aging or alter aberrant energy management in LOAD. Nicotinamide riboside might still be of value in combination with other agents in preventive or therapeutic intervention strategies to address the aging process or age‐associated dementia.

## Graphical abstract

Nicotinamide riboside (NR) and caffeine increase nicotinamide adenine dinucleotide (NAD), transiently modulate bioenergetic functions, and change the expression of NAD‐synthesizing, recycling, or degrading genes in dermal fibroblasts or induced pluripotent stem cell (iPSC)‐derived neural progenitors and astrocytes from late‐onset Alzheimer's disease (LOAD) patients and healthy control individuals, but do not alter the characteristics of an inherent LOAD‐associated bioenergetic phenotype.

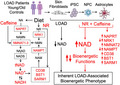

**Abbreviations**
ATP: adenosine triphosphate
BST1: bone marrow stromal cell antigen‐1
CAC: citric acid cycle
ECAR: extracellular acidification rate
ETC: electron transfer chain
FAD: flavin adenine dinucleotide
FCCP: carbonyl cyanite‐4 (trifluoromethoxy) phenylhydrazone
G3P: glycerol‐3‐phosphate
iPSC: induced pluripotent stem cell
LOAD: late‐onset Alzheimer's disease
MAS: malate–aspartate shuttle
NA: nicotinic acid
NAAD: nicotinic acid adenine dinucleotide
NAD: nicotinamide adenine dinucleotide
NADS: NAD synthase
NAPRT: nicotinic acid phosphoribosyltransferase
NMN: nicotinamide mononucleotide
NMNAT: nicotinamide mononucleotide transferases
NPC: neural progenitor cell
NR: nicotinamide riboside
NRK1: nicotinamide riboside kinase 1
OC: old control
OCR: oxidative consumption rate
OxPhos: oxidative Phosphorylation
PARP: poly (ADP‐ribose) polymerase 1
PER: proton efflux rate
PPP: pentose phosphate pathway
RR: redox ratio
SARM: sterile‐alpha and TIR motif containing 1
SCR: spare respiratory capacity
SIRT: sirtuins
YC: young control

## 1 INTRODUCTION

LOAD, the most common form of dementia, is an age‐related neurodegenerative disorder associated with neuronal dysfunction and death (Sengoku, 2020; Soria Lopez et al., 2019). LOAD is characterized by a combination of several interacting pathological processes, some of which are commonly seen with age (Amtul, 2016; Harrison & Owen, 2016; Herrup, 2015; Mattson & Arumugam, 2018; Sengoku, 2020; Soria Lopez et al., 2019). A key factor is altered bioenergetics, that is, the metabolism of fuel molecules to produce and utilize energy through glycolysis, mitochondrial respiration, and the pentose phosphate pathway (PPP), which together produce ATP and essential metabolites. Glycolysis produces pyruvate and lactate from glucose and reduces NAD, an oxidizing agent in redox reactions and mitochondrial electron transfer. Mitochondria metabolize carbohydrates (pyruvate or lactate), ketone bodies, fatty acids, glutamine, and other molecules, processed through the Krebs or tricarboxylic (citric) acid cycle (TCA, CAC) and oxidative phosphorylation (OxPhos), which depends, in turn, on redox reactions involving NAD and FAD. PPP leads to reduction of NAPD^+^ to NAPDH, used for fatty acid biosynthesis and regeneration of reduced glutathione. Many brain disorders, including LOAD, show evidence of abnormal bioenergetics, which may lead to diminished neuroprotective capacities, enhancing and accelerating age‐related neuronal decline (Jove et al., 2014; Mattson & Arumugam, 2018; Ryu, Bormann, et al., 2021; Ryu, Cohen, et al., 2021; Sun et al., 2016; Swerdlow & Khan, 2004).

**FIGURE 1:**
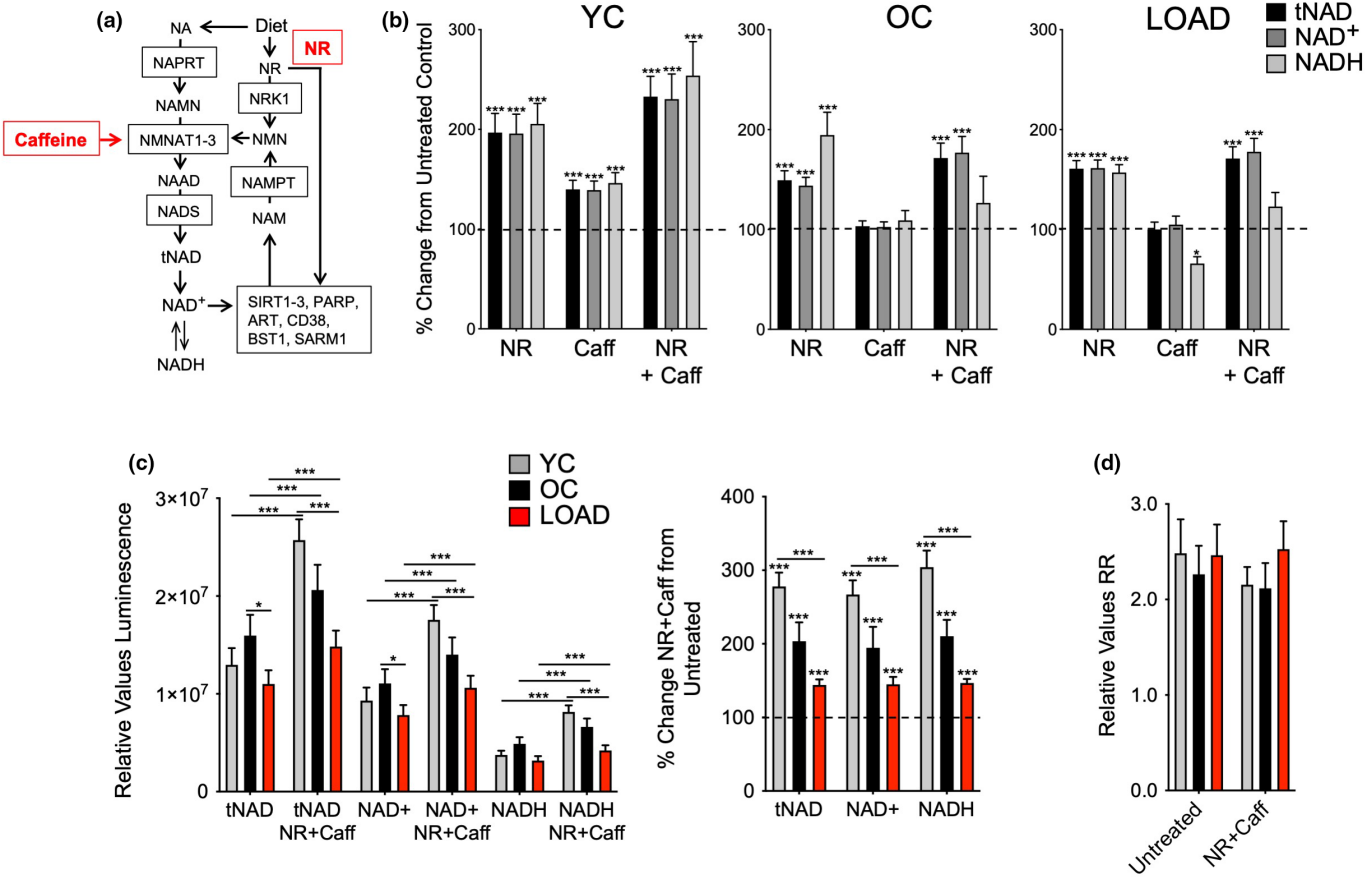
NR, caffeine, or NR+Caff increase NAD co‐factor availability in human skin fibroblasts. (a) Schematic of NAD^+^ de novo synthesis or recycling. Dietary products, including nicotinic acid (NA) and nicotinamide riboside (NMR or NR), are converted to nicotinic acid mononucleotide (NAMN) or nicotinamide mononucleotide (NMN) by nicotinic acid phosphoribosyltransferase (NAPRT) or nicotinamide riboside kinase 1 (NMRK1 or NRK1), respectively. NAMN and NMN are substrates for nicotinamide mononucleotide transferases subtypes 1–3 (NMNAT1‐3) to produce the NAD precursor nicotinic acid adenine dinucleotide (NAAD) which is converted to NAD by NAD synthase (NADS). Oxidized NAD (NAD^+^) or NR are co‐substrates for SIRTs, PARPs, ARTs, CD38, BST1, and SARM1, and consumed to nicotinamide (NAM) which is recycled to NMN by nicotinamide phosphotransferase (NAMPT). Caffeine has been proposed to induce the transcription of NMNAT2 (Ali et al., 2017). (b) Changes of tNAD, NAD^+^, and NADH in skin fibroblasts from a YC (21 years), OC (65 years), and LOAD (76 years) sample after treatment with NR, caffeine, or NR+Caff for 24 hrs plotted as percent change from untreated controls. (c) tNAD, NAD^+^, and NADH in YC (*n* = 5, average age 25), OC (*n* = 8, average age 67), and LOAD (*n* = 8, average age 70) fibroblasts in untreated or NR+Caff treated cells for 24 h plotted as relative values luminescence (left panel) or as percent change NR+Caff over untreated condition (right panel). (d) Calculated RR. Data are means ± SEM from two repeat experiments. *p < 0.1; **p < 0.05; ***p < 0.01 using one‐way ANOVA, depicting significant changes between treated and untreated cells or groups

Nicotinamide adenine dinucleotide is crucial in all these pathways. Its reduced form NADH plays a critical role as an energy‐transfer intermediate and is an important metabolite of the mitochondrial electron transfer chain (ETC), where it is oxidized to NAD^+^, donating electrons and protons during respiration. NAD concentration is determined by new synthesis and recycling (Figure 1a and reviewed in [Canto et al., 2015; Covarrubias et al., 2021; Verdin, 2015]). NAD levels, and factors required for its synthesis and/or recycling, such as nicotinamide mononucleotide adenylyl transferases (NMNAT), diminish with age and in neurodegenerative diseases, including LOAD (Ali et al., 2016; Covarrubias et al., 2021; Lautrup et al., 2019; Ryu, Bormann, et al., 2021; Ryu, Cohen, et al., 2021; Sonntag et al., 2017; Verdin, 2015; Zhu et al., 2015) Because reduced NAD^+^ recycling impairs glycolysis and OxPhos and enhanced NAD^+^ metabolism can protect neurons from degeneration, NAD has been suggested as a therapeutic agent in neurodegenerative diseases (Braidy & Liu, 2020b; Covarrubias et al., 2021; Lautrup et al., 2019; Liu et al., 2008; Verdin, 2015).

Both supplemental nicotinamide riboside (NR) and nicotinamide mononucleotide (NMN) have been identified as possible therapeutic candidates for neurodegenerative diseases (Braidy & Liu, 2020a; Brakedal et al., 2022; Mehmel et al., 2020). Nicotinamide riboside, a naturally occurring form of vitamin B3, is commercially available, as a nutraceutical (Dellinger et al., 2017). It is converted to NMN by NR kinase 1 (NRK1), then processed to the NAD precursor nicotinic acid adenine dinucleotide (NAAD) by NMNAT1, 2, or 3 (Figure 1a) (Bieganowski & Brenner, 2004; Ratajczak et al., 2016). Treatment with NR upregulates intracellular and mitochondrial NAD^+^ levels and expression or function of sirtuins (SIRT), which are NAD^+^‐dependent protein deacetylases involved in many cellular functions and specifically implicated in aging and neurodegeneration (Belenky et al., 2007; Sasaki et al., 2006; Satoh et al., 2017; Schondorf et al., 2018; Verdin, 2015). NAD^+^ is also a co‐substrate for poly (ADP‐ribose) polymerase1 (PARP‐1), a DNA repair enzyme that consumes large amounts of NAD^+^ for DNA repair, a process altered in various brain disorders (Cohen, 2020; Pehar et al., 2018). In AD transgenic mice, administration of NMN improves mitochondrial bioenergetics (Long et al., 2015), and treatment with NR elevates brain NAD^+^ levels, improves cognition, reduces beta‐amyloid (Aβ) accretion, upregulates mitochondrial gene expression, increases the degradation of Peroxisome proliferator‐activated receptor (PPAR) gamma coactivator 1‐alpha (PGC‐1α) (Gong et al., 2013), and lessens *p*tau pathology, DNA damage, neuroinflammation, cell senescence, and apoptosis of hippocampal neurons, while increasing SIRT3 activity, hippocampal synaptic plasticity, and cognitive functions (Hou et al., 2018, 2021).

NMNAT2 may also play a role in neuroprotection (Ali et al., 2016; Gerdts et al., 2016; Lavado‐Roldan & Fernandez‐Chacon, 2016). It is suppressed by MAPK signaling which can lead to axon degeneration (Walker et al., 2017). The expression of NMNAT2 is downregulated in AD patients' brains (Ali et al., 2016), and caffeine (1,3,7‐trimethylxanthine) increases the expression of NMNAT2 in cortex of wild type, NMNAT2 heterozygous knockout, and rTg4510 tauopathy mice (Ali et al., 2017). Caffeine, found in many beverages, passes the blood–brain barrier, has acute and stimulating effects in the central nervous system, and may have protective effects against dementia, and LOAD, in particular (Eskelinen & Kivipelto, 2010; McCall et al., 1982; Nehlig, 1999). Physiologic doses of caffeine induce mitochondrial biogenesis and protection and enhance oxidative metabolism (Schnuck et al., 2018).

We previously observed reductions of NAD^+^ and NADH along with other bioenergetic alterations in cells from LOAD patients, including skin fibroblasts and iPSC‐derived NPCs and astrocytes (Ryu, Bormann, et al., 2021; Ryu, Cohen, et al., 2021; Sonntag et al., 2017). Here, we hypothesized that treatment with NR and caffeine might restore NAD levels, and “correct” the bioenergetic profiles of LOAD cells.

## 2 RESULTS

### 2.1 NR and caffeine boost NAD in fibroblasts, NPCs, and astrocytes

We established the experimental conditions to elevate NAD by NR and caffeine in different human cell lines (data not shown) and in context of LOAD or age by using fibroblasts derived from a LOAD patient (age 76), and a young (YC, age 21) and an old (OC, age 65) healthy control individual, as previously described (Ryu, Cohen, et al., 2021; Sonntag et al., 2017; Figure 1b). Twenty four‐hour treatment with 2 mM NR significantly increased NAD^+^, NADH, and total (t)NAD (NAD^+^ plus NADH) in YC, OC, and LOAD fibroblasts with the strongest effect in YC cells. An optimal dose of 200 μM caffeine significantly increased the redox co‐factors in YC cells, however, to lower levels than NR, but did not boost levels in OC or LOAD cells. A combination of both NR and caffeine (NR+Caff) produced the highest increases of tNAD, NAD^+^, and NADH; it was used in all subsequent treatment experiments.

We tested NR+Caff in additional YC, OC, and LOAD fibroblasts confirming an increase in NAD 24 h after treatment with the highest response rates in YC and the lowest in LOAD cells (Figure 1c). In untreated conditions, we confirmed our previous findings of slightly increased tNAD, NAD^+^, and NADH in OC but decreased levels in LOAD cells (Ryu, Cohen, et al., 2021; Sonntag et al., 2017). Redox ratios (RR) showed slight decreases in YC and OC, but not in LOAD cells after treatment with NR+Caff (Figure 1d). Pearson coefficient analyses showed strong positive correlations of NAD^+^ and NADH in all cells and conditions (Figure S1a).

**FIGURE 2:**
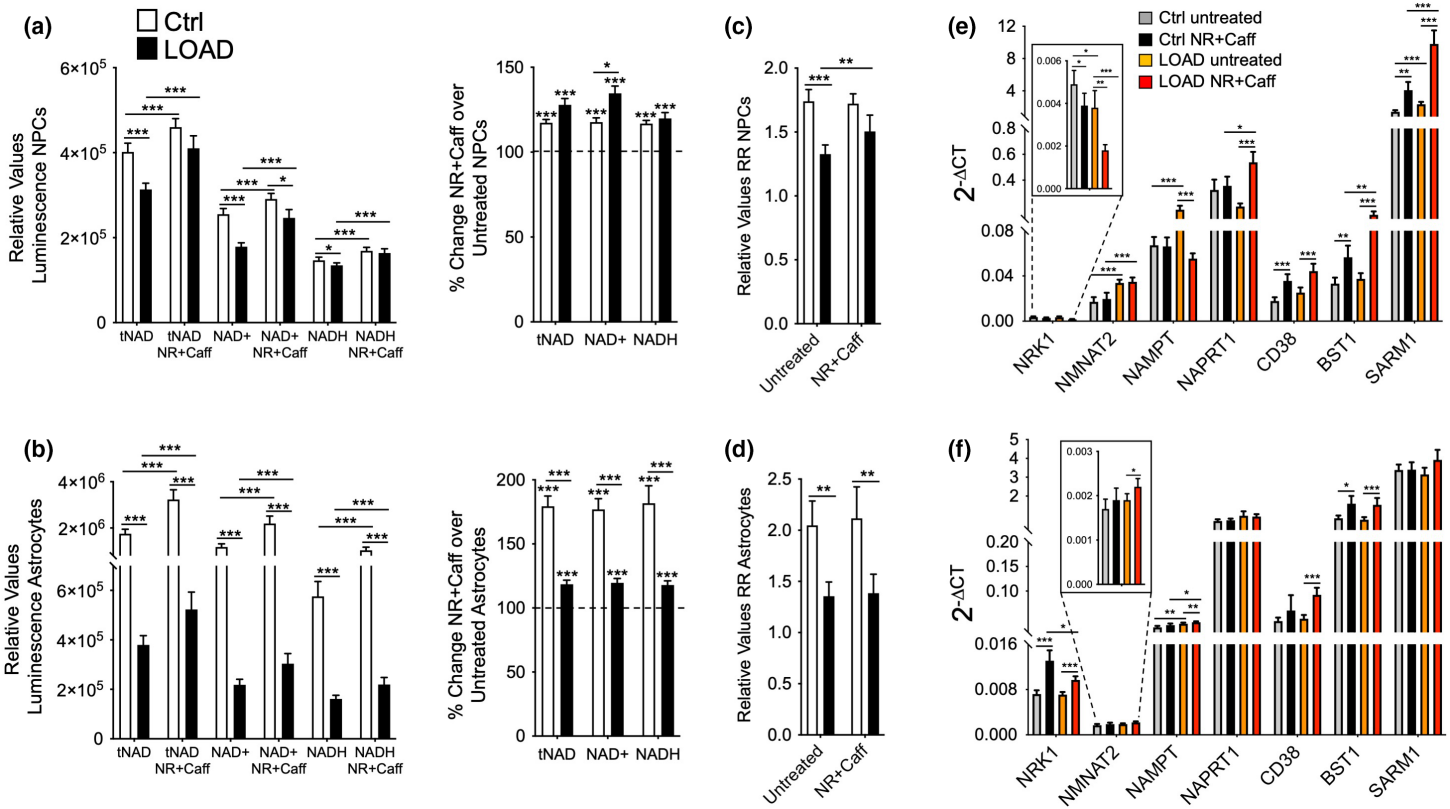
NR+Caff increases NAD redox factors in NPCs and astrocytes and affects the expression of genes that are involved in NAD metabolism. (a, b) tNAD, NAD^+^, and NADH levels in control or LOAD iPSCs‐derived NPCs (a) and astrocytes (b) in untreated or NR+Caff treated cells for 24 h plotted as relative values luminescence (left panels) or as percent change NR+Caff over untreated condition (right panels). (c, d) calculated RR in NPCs (c) and astrocytes (d). (e, f) gene expression of NRK1, NMNAT2, NAMPT, NAPRT1, CD38, BST1, and SARM1 measured by qRT‐PCR and plotted as 2^−ΔCT^ values in NPCs (e) and astrocytes (f). Data are from *n* = 9 samples in each group and means ± SEM from two repeat experiments. *p < 0.1; **p < 0.05; ***p < 0.01 using one‐way ANOVA, depicting significant changes between treated and untreated cells or groups

We supplemented NPCs and astrocytes differentiated from healthy control‐ or LOAD patients'‐derived iPSC lines (Ryu, Bormann, et al., 2021; Ryu, Cohen, et al., 2021) with NR+Caff, which increased tNAD, NAD^+^, and NADH in both LOAD and Control NPCs and astrocytes at 24 h post‐treatment with stronger effects in LOAD NPCs than LOAD astrocytes, versus control cells (Figure 2a,b). While the redox factors in LOAD NPCs could be increased to nearly the same levels as control cells, LOAD astrocytes still had significantly lower tNAD, NAD^+^, and NADH than control astrocytes after supplementation with NR+Caff. Diminished RR in LOAD NPCs and astrocytes was slightly increased in LOAD NPCs post NR+Caff treatment but not in LOAD astrocytes (Figure 2c,d). There were strong positive correlations between NAD^+^ and NADH in all cell types and conditions (Figure S1b).

Collectively, the data demonstrate that an age or LOAD background limits the ability to boost the NAD pool by NR+Caff in fibroblasts and astrocytes, while NPCs with a LOAD background had enhanced co‐factor production. Consistent with previous findings (Ryu, Bormann, et al., 2021; Ryu, Cohen, et al., 2021), all cell types exhibit positive RRs which are lower in LOAD NPCs and astrocytes but higher in LOAD fibroblasts indicating that a LOAD background is associated with increased reducing activity in brain‐like cells and enhanced oxidative capacity in skin cells. NR+Caff does not change this LOAD pattern in fibroblasts and astrocytes but increases oxidation in LOAD NPCs. The strong positive NAD^+^ and NADH correlations in all cells and conditions demonstrate that despite differences in RR, the overall redox capacity in fibroblasts, NPCs, and astrocytes is not affected by age or LOAD background.

### 2.2 Expression of genes for NAD metabolism is altered in LOAD and in response to NR+Caff

Our previous studies showed that some genes in the NAD synthesis and recycling pathway or NAD‐consuming enzymes are deregulated in LOAD cells (Ryu, Bormann, et al., 2021; Ryu, Cohen, et al., 2021; Sonntag et al., 2017). We assessed the effects of NR+Caff in NPCs and astrocytes on the transcription of NRK1, NMNAT2, NAMPT, NAPRT, and CD38, CD157 (BST1), and SARM1, which have been implicated in aging, neurodegeneration, and AD (see Figure 1a, and summarized in [Covarrubias et al., 2021; Verdin, 2015]). There were distinct gene expression profiles in NPCs and astrocytes, and we confirmed our previous observation of downregulated NRK1 and upregulated NMNAT2 and NAMPT expression in untreated LOAD NPCs, and an upregulation of NAMPT in LOAD astrocytes (Figure 2e,f). NR+Caff reduced NRK1 and NAMPT but increased NAPRT1 expression in Control or LOAD NPCs, while in astrocytes the expression of NRK1, NMNAT2, and NAMPT2 were increased. As for the NAD‐consuming molecules CD38, BST1, and SARM1, expression levels were not different between untreated Control and LOAD NPCs or astrocytes; however, except for SARM1 in astrocytes, gene expression was significantly upregulated after NR+Caff treatment.

### 2.3 NR+Caff increases bioenergetic functions in NPCs and decreases them in astrocytes and fibroblasts

**FIGURE 3:**
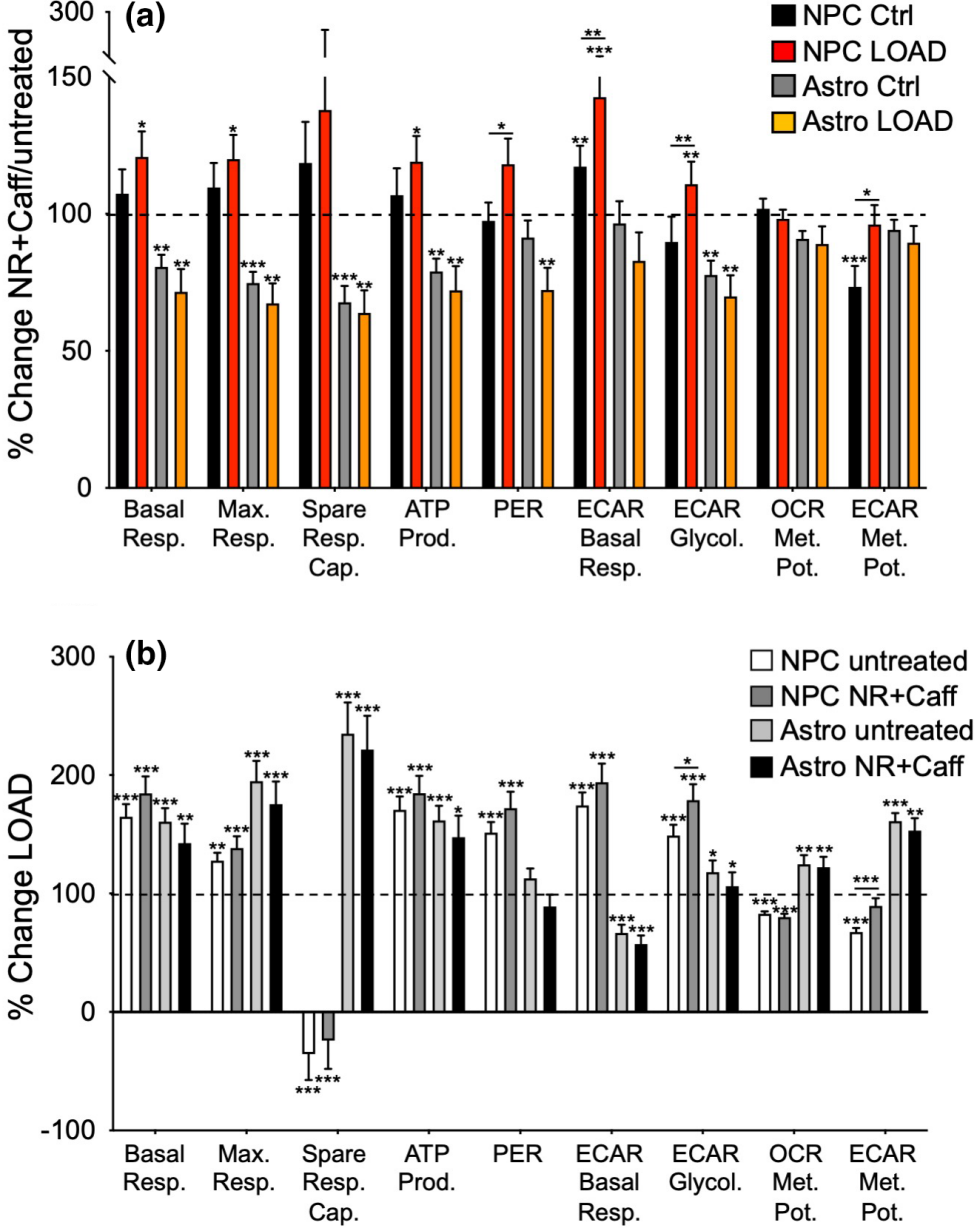
NR+Caff increases bioenergetic functions in NPCs and decreases them in astrocytes. (a) Calculated values of OCR, ECAR, and the metabolic potentials ([stressed OCR or ECAR/baseline OCR or ECAR] x 100%)) from control (*n* = 9) and LOAD NPCs (*n* = 9) and control (*n* = 9) and LOAD astrocytes (*n* = 9) plotted as percent change after treatment with NR+Caff for 24 h. (b) the same data as shown in (a) plotted as percent change LOAD over controls in untreated and NR+Caff treated conditions. Data are means ± SEM from two repeat experiments. *p < 0.1; **p < 0.05; ***p < 0.01 using one‐way ANOVA, depicting significant changes between treated and untreated cells or groups

We assessed effects of NR+Caff treatment on mitochondrial respiration and glycolysis in LOAD and Control NPCs and astrocytes using Seahorse assays as previously reported (Ryu, Bormann, et al., 2021; Ryu, Cohen, et al., 2021; Sonntag et al., 2017). LOAD NPCs had increased respiration, glycolysis, and energy (ATP) output in baseline conditions, and diminished respiratory or glycolytic functions in response to mitochondrial stress (Figure S2a–e). NR+Caff slightly increased oxidative consumption (OCR) both in Control and LOAD NPCs, reflecting an increase in mitochondrial basal and maximal respiration, ATP production, proton efflux rate (PER), and spare respiratory capacity (SRC; Figure 3a). Spare respiratory capacity is calculated as the difference between maximal and basal respiration, reflecting the ability to respond to increased energy demand by producing additional ATP through OxPhos. As previously demonstrated (Ryu, Bormann, et al., 2021; Ryu, Cohen, et al., 2021), LOAD NPCs increased basal respiration and had negative SRC values (Figure 3b, Figure S2b). NR+Caff did not change this pattern; neither NR nor caffeine improved the diminished respiration in LOAD. NR+Caff also increased the baseline extracellular acidification rate (ECAR), an indicator of glycolysis and lactate production. However, mitochondrial stress‐induced glycolysis and OCR/ECAR indexes for basal respiration and ATP production, reflecting mitochondrial energy output relative to glycolysis, were unchanged or decreased (Figure 3a, Figure S2c). Additional data calculations using Seahorse “Cell Energy Phenotype Tests” showed slight increases of OCR and significant increases of ECAR levels at baseline or in stress conditions after treatment with NR+Caff both in Control and LOAD NPCs, but unchanged or decreased OCR and ECAR metabolic potentials, as measures of the relative respiratory or glycolytic response to mitochondrial stress (percent stress over baseline condition; Figure 3a, Figure S2d,e). Thus, except for a significant increase in glycolysis, the overall bioenergetic profile associated with LOAD, as previously described (Ryu, Bormann, et al., 2021; Ryu, Cohen, et al., 2021), was not changed by NR+Caff (Figure 3b). However, consistent with treatment‐induced higher NAD levels, LOAD NPCs had slightly better bioenergetic responses than Control cells.

As previously reported (Ryu, Bormann, et al., 2021; Ryu, Cohen, et al., 2021), LOAD astrocytes experienced increased mitochondrial respiration, glycolysis, and energy (ATP) output in baseline conditions and increased respiratory or glycolytic functions in response to mitochondrial stress compared to Control cells (Figure S3a‐e). However, in contrast to NPCs, when treated with NR+Caff, Control and LOAD astrocytes exhibited a stark reduction of both respiratory (basal and maximal respiration, SRC, ATP production, PER) and glycolytic (ECAR basal respiration, glycolysis) measures (Figure 3a, Figure S3b,c). Consistently, the baseline and stress OCR and ECAR levels and the OCR and ECAR response rates to mitochondrial stress were reduced (Figure 3a, Figure S3d,e). Overall, the reduction of respiration and glycolysis in NR+Caff treated LOAD astrocytes reached levels like in untreated astrocytes; however, as in NPCs, the LOAD‐associated bioenergetic profile was not changed (Figure 3b). Also, in contrast to LOAD NPCs and consistent with lower NAD levels, LOAD astrocytes had slightly lower bioenergetic responses to NR+Caff than Control cells. To substantiate the results in iPSC‐derived astrocytes, we analyzed secondary human astrocytes and saw similar diminished bioenergetic functions in response to NR + Caff (Figure S3f).

**FIGURE 4:**
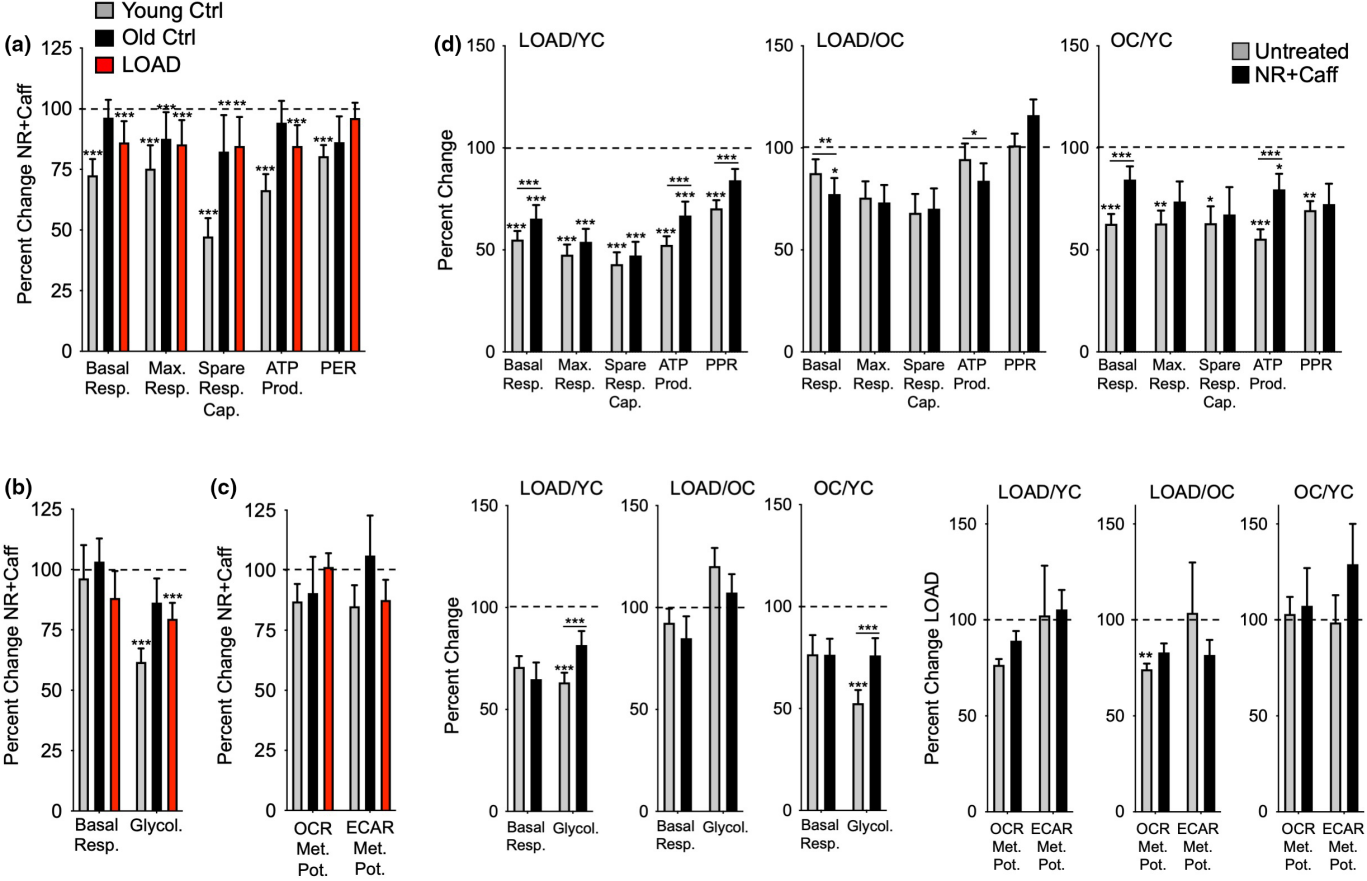
NR+Caff decreases bioenergetic functions in fibroblasts. (a–c) calculated values of OCR (a), ECAR (b), and metabolic potentials ([stressed OCR or ECAR/baseline OCR or ECAR] x 100%)) (c). Data are from YC (*n* = 17, average age 37), OC (*n* = 11, average age 66), and LOAD (*n* = 10, average age 71) fibroblasts and plotted as percent change after treatment with NR+Caff for 24 h. (d) the same data as shown in (a–c) plotted as percent change LOAD/YC, LOAD/OC, and OC/YC in untreated and NR+Caff treatment conditions. Data are means ± SEM from two repeat experiments. *p < 0.1; **p < 0.05; ***p < 0.01 using one‐way ANOVA, depicting significant changes between treated and untreated cells or groups

We also analyzed fibroblasts and found that all cells, regardless of age or disease, had reduced respiration and glycolysis after treatment with NR+Caff (Figure 4a‐c, Figure S4), like the iPSC‐derived astrocytes. These effects appeared to be more pronounced in YC than in OC or LOAD cells. NR+Caff did not substantially change the reduction of LOAD‐ and OC‐associated bioenergetic functions when compared to YC cells (Figure 4d). There were some increases in respiration and glycolysis in LOAD and OC fibroblasts compared to YC cells but not in LOAD cells compared to OC cells, indicating higher bioenergetic plasticity in age in response to NR+Caff than in LOAD.

To evaluate a potential impact of NR+Caff on cell growth, we summarized the CyQuant measurements used to normalize Seahorse values. There was a decrease of cell fluorescence in NR+Caff treated NPCs and astrocytes which was significant in NPCs but not in astrocytes and more pronounced in LOAD NPCs than in controls (Figure S5a,b). In contrast, fluorescence was increased in YC and OC fibroblasts but not in LOAD cells (Figure S5c). These data indicate that under the experimental conditions used here, the viability of NPCs and astrocytes decreased or were unchanged during the 24‐h NR+Caff treatment, while control fibroblasts still proliferated. In addition, a negative effect of NR+Caff on cell viability or growth was stronger in LOAD cells.

### 2.4 NR+Caff alters the processing of bioenergetic metabolites in NPCs, astrocytes, and fibroblasts

We determined the capacity of NPCs, astrocytes, and fibroblasts to process bioenergetic metabolites using the Biolog MitoPlate S‐1 platform as described in (Ryu, Bormann, et al., 2021; Ryu, Cohen, et al., 2021) and in Material and Methods. In Control NPCs, there was an overall increase in metabolism of substrates involved in oxidative respiration, including CAC, ETC, malate–aspartate shuttle (MAS), and β‐oxidation, and amino acid processing, which lasted for almost 24 h. However, metabolism of some substrates of glycolysis or PPP, such as glycogen, D‐gluconase‐6‐PO4, and D‐gluconate‐6‐PO4, along with L‐lactic acid, a key substrate connecting glycolysis with oxidative respiration, D,L‐α‐glycerol‐PO4, the substrate of the glycerol‐3‐phosphate shuttle (G3P), and D,L‐isocitric and cis‐asconitic acids in the CAC, were reduced. LOAD NPCs had different patterns, showing slight increases in all oxidative processes in the first hour after NR+Caff treatment, which persisted or were restored to baseline levels for substrates metabolized in β‐oxidation, MAS, G3P, or as amino acids, while metabolites in CAC, ETC, and L‐glutamine were reduced. Compared to Control NPCs, LOAD NPCs had no changes in the metabolism of glycolytic substrates but showed similar increases in D,L‐β‐hydroxy‐butyric acid processing. Overall, LOAD NPCs experienced lesser responses to NR+Caff than Control cells. Both Control and LOAD astrocytes demonstrated an increase of glycolytic and respiratory functions immediately after NR+Caff treatment that lasted for about 8 h in Control and 6 h in LOAD astrocytes, after which the cells starkly reversed their bioenergetic functions, which eventually declined to lower levels than seen in untreated Control cells. As in NPC lines, the dynamic responses to NR+Caff appeared to be less pronounced in LOAD astrocytes than Control cells. Similar observations were made in secondary human astrocytes, indicating an astrocytic‐specific response pattern to NR and caffeine treatment. In fibroblasts, both YC and OC had initial increases in metabolic functions, for up to 3 h, followed by a decline in processing most of the substances, except for amino acids. In contrast, LOAD fibroblasts did not appear to be responsive to NR+Caff at all. The data from the Biolog experiments were consistent with the bioenergetic profiles from Seahorse experiments at 24 h post NR+Caff treatment, demonstrating slight increases of metabolic functions in NPCs and decreases in astrocytes and fibroblasts.

### 2.5 Bioenergetic responses to NR+Caff are transient

**FIGURE 5:**
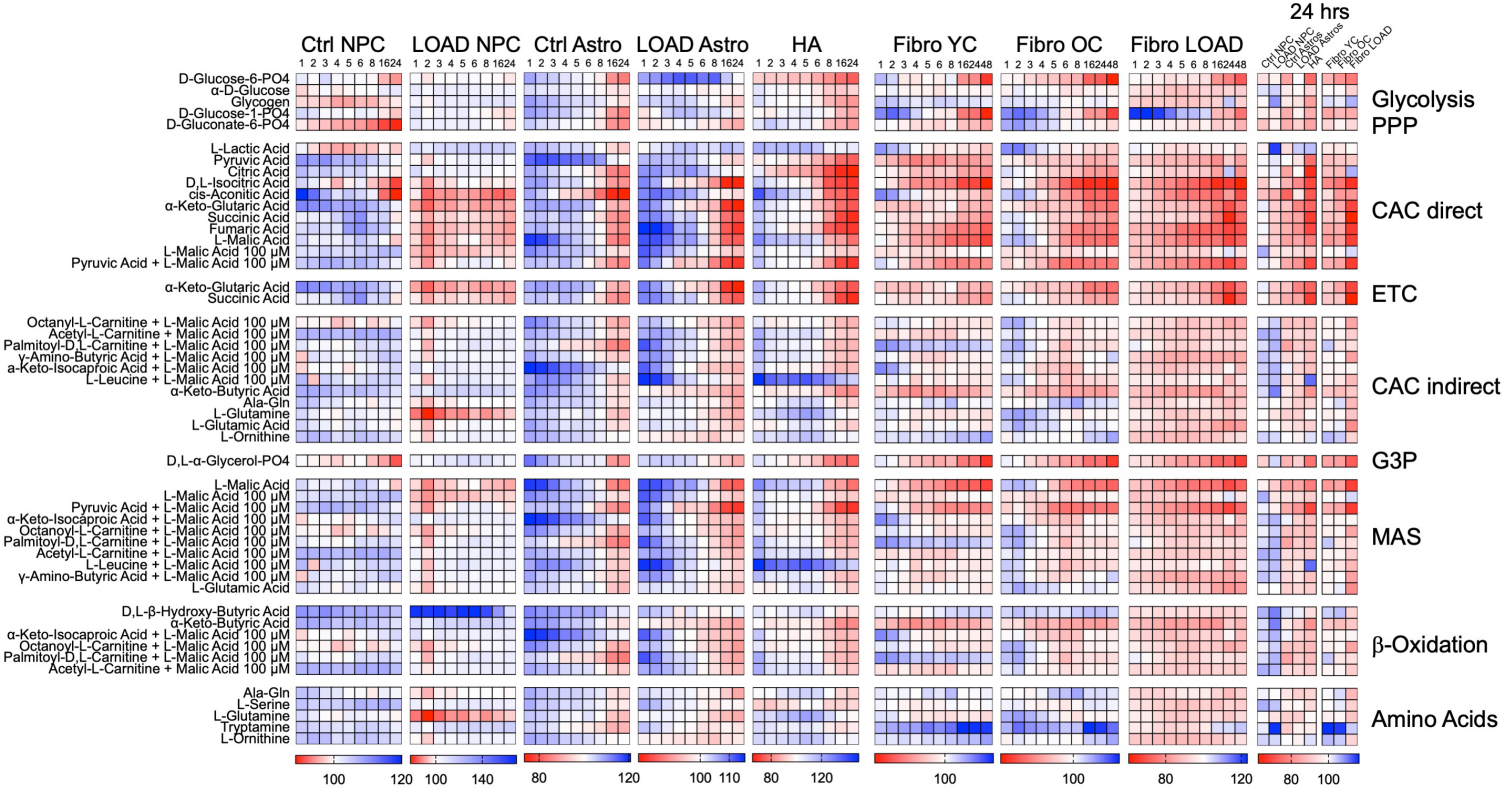
NR+Caff alters bioenergetic substrate metabolism in NPCs, astrocytes, and fibroblasts. Heatmaps of data from biolog experiments on control (*n* = 9) and LOAD (*n* = 9) NPCs or astrocytes, human secondary astrocytes (HA), and YC (*n* = 7, average age 30), OC (*n* = 9, average age 71), and LOAD (*n* = 7, average age 73) fibroblasts for kinetic measurements. Cells were supplemented with NR+Caff and OD 590 was measured at 1, 2, 3, 4, 5, 6, 8, 16, 24, and 48 h during culture. Data are plotted as percent change treated versus untreated cells (the measurement at 24 h is additionally plotted at the far right summarizing all samples at the same percent scale). Metabolites are clustered according to their processing in bioenergetic pathways as explained in the text and published elsewhere (Ryu, Bormann, et al., 2021; Ryu, Cohen, et al., 2021). Red indicates percent decrease and blue percent increase

**FIGURE 6:**
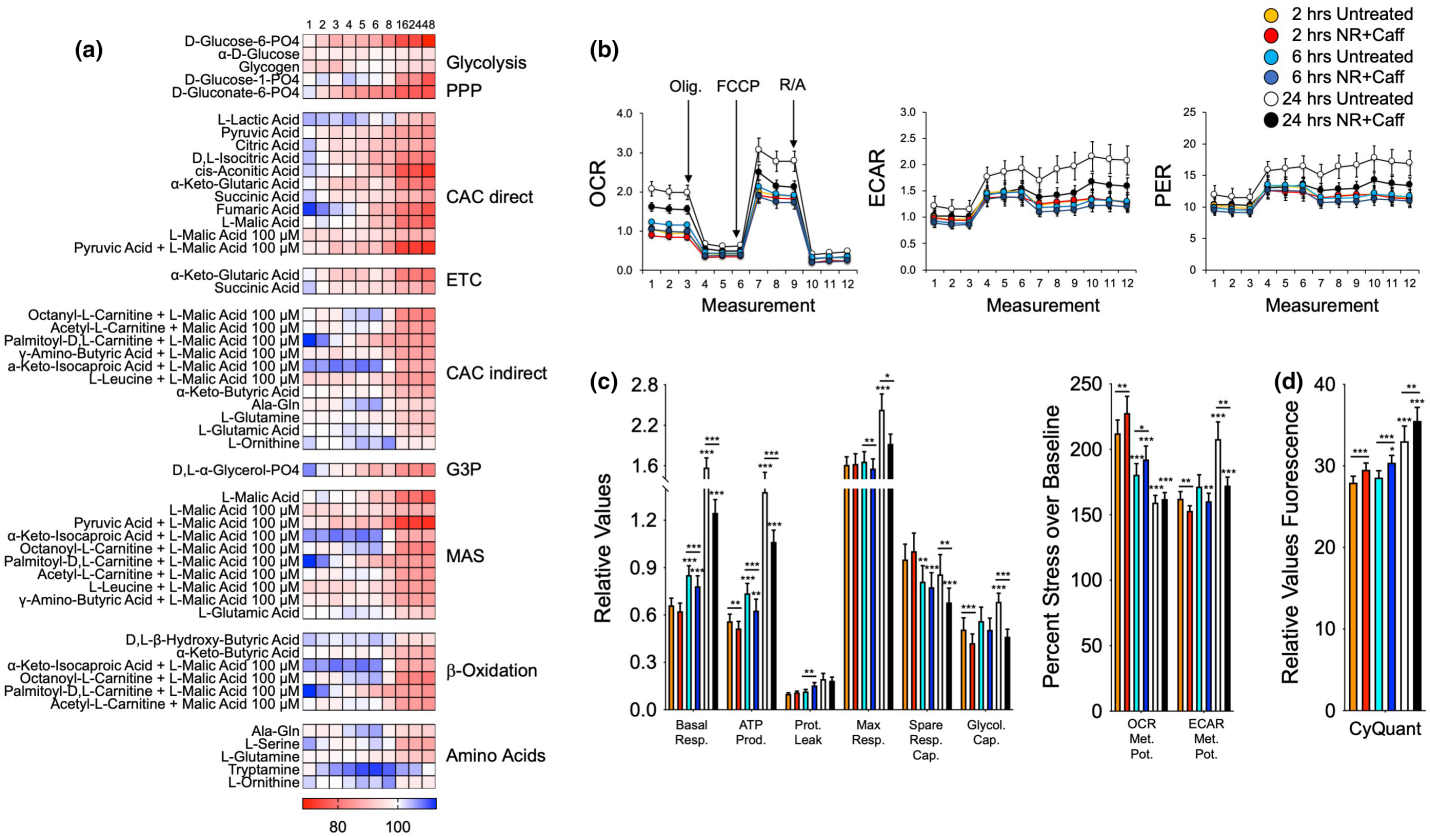
NR+Caff kinetically changes bioenergetic functions in HFF1 fibroblasts. Heatmaps of data from biolog experiments (a) and Seahorse experiments (b‐d) on HFF1 cells treated with NR+Caff for 2, 6, and 24 h. Shown are percent changes in NR+Caff treated versus untreated cells at 1, 2, 3, 4, 5, 6, 8, 16, 24, and 48 h for metabolite processing (a) and values derived from Seahorse analysis (b and c) or CyQuant measurements (d). Data are means ± SEM from two (a) or six (b‐d) repeat experiments. *p < 0.1; **p < 0.05; ***p < 0.01 using one‐way ANOVA, depicting significant changes between 6 and 24 h compared to 2 h measurements and treated versus untreated cells

To substantiate the observed dynamic bioenergetic responses, we treated HFF1 fibroblasts with NR+Caff and conducted Biolog and Seahorse experiments at 2, 6, and 24 h post‐treatment (Figure 6). Newborn foreskin HFF1 cells had patterns of bioenergetic substrate metabolism like the adult subjects'‐derived fibroblast cell lines and responded in the same kinetic fashion to NR+Caff seen in YC and OC cells (Figure 6a). In Seahorse experiments, there was an increase of respiration and glycolysis and a decrease of respiratory plasticity at 6 and 24 h compared to 2 h cell growth (Figure 6b,c). Surprisingly, no significant increases but, rather, reductions of respiratory and glycolytic functions were observed after NR+Caff supplementation. However, OCR metabolic potentials were significantly increased, while the ECAR metabolic potentials were decreased, indicating that NR+Caff enhanced the cells' respiratory but not glycolytic response to mitochondrial stress (Figure 6c, right panel). In addition, CyQuant data showed that as seen in YC or OC fibroblasts, NR+Caff treatment had an apparent positive effect on cell viability and growth (Figure 6d). Altogether, the data in Figures 5 and 6 demonstrate that, over time, substrate metabolism in fibroblasts is largely concomitant with the cells' dynamic respiratory and glycolytic functions in response to NR+Caff, and that fibroblasts have kinetic patterns that are independent of age, while the relative unresponsiveness of LOAD fibroblasts is disease specific.

## 3 DISCUSSION

Changes in energy metabolism are hallmarks of brain aging, and there is substantial evidence that altered metabolic functions, including the production and use of energy with age, lead to neural vulnerability and diminished neuroprotection, accelerating neurodegenerative brain disorders, including LOAD (Jove et al., 2014; Mattson & Arumugam, 2018; Sun et al., 2016; Swerdlow & Khan, 2004). Our present and previously published studies suggest that LOAD cells have alterations in energy production due to impairments in bioenergetic substrate uptake or metabolism, including uptake of glucose and generation and maintenance of redox factors, and a disconnect between glycolytically generated NADH and the G3P shuttle system. They also have enhanced alternative pathways for energy production, such as β‐oxidation, amino acid metabolism, and transport of reducing agents with MAS (Ryu, Bormann, et al., 2021; Ryu, Cohen, et al., 2021; Sonntag et al., 2017). Overall, it appears that LOAD cells are differentially metabolically programmed, exhibiting a LOAD‐associated bioenergetic phenotype, with LOAD cells “working harder” to produce and maintain energy balance in both baseline conditions and under stress. This phenotype already appears in LOAD iPSC‐derived NPCs and may, thus, be an inherent risk factor for altered aging and the development of dementia. In other words, altered bioenergetics is likely an early factor of cellular vulnerability that negatively impacts response to age‐ and disease‐associated stress, leading to an aberrant aging process and the typical pathophysiological characteristics of dementia and LOAD later in life (Ryu, Cohen, et al., 2021).

Here, we assessed if reduced levels of NAD in LOAD cells and associated bioenergetic alterations can be restored with NR and caffeine. Our data demonstrate that NR and/or caffeine treatment, alone or in combination, can dose‐dependently and cell‐specifically elevate the NAD pool in several human cell types. The increases appear to be driven more by NR than caffeine, though caffeine can enhance the effects of NR. Treatment with NR and caffeine demonstrates that fibroblasts from old individuals and even more so from LOAD patients have lesser responses and lower tNAD, NAD^+^, and NADH levels achieved than those from young individuals. LOAD astrocytes had reduced redox factors in both untreated and treated conditions. However, LOAD NPCs demonstrated higher responses than Control NPCs and reached NAD levels like those seen in untreated Controls. Thus, both age and a LOAD background not only determine NAD levels but also the response to NAD‐boosting molecules like NR and caffeine, which appears to be diminished in mature and old cells and enhanced in immature or young cells. Reduced availability of NAD co‐factors can be a consequence of differentially expressed genes in NAD synthesis and recycling, or consumption. Treatment with NR and caffeine differentially affected gene expression in NPCs and astrocytes, for example, the NR‐processing enzyme NRK1 was upregulated in astrocytes but downregulated in NPCs, and in LOAD NPCs NAMPT was upregulated but NAPRT was downregulated, while LOAD astrocytes increased NMNAT2 and NAPRT, indicating cell‐specific and disease‐associated responses. The similar expression levels of CD38, BST1, and SARM1 in untreated Control and LOAD cells suggest that enzymatic NAD degradation may not be a reason for a diminished NAD pool in LOAD. However, their upregulation in response to NR and caffeine could point to increased enzymatic NR and NAD degradation contributing to the observed alterations in bioenergetic functions (Angeletti et al., 2022; Figley et al., 2021; Yaku et al., 2021). In addition, these enzymes have been implicated in neurodegeneration by inducing, for example, senescence, (neuro)inflammation, axon degeneration, and other effects (summarized in [Covarrubias et al., 2021; Verdin, 2015]) suggesting that supplementation with NR and caffeine could also contribute to enhancing neurodegenerative processes.

Bioenergetic changes in response to NR and caffeine were also cell‐specific and to some extent LOAD‐associated. While control NPCs had increased bioenergetic functions in the mitochondrial respiratory chain, such as CAC, ETC, MAS, β‐oxidation, and amino acid metabolism that lasted for at least 16 h, these effects were less pronounced in LOAD NPCs, which had reductions in CAC, ETC, MAS, and L‐glutamine metabolism. The features occurred despite slight increases in respiration and glycolysis in LOAD NPCs as measured by the Seahorse experiments, which could be a consequence of their higher response rates to NR and caffeine and increased NAD levels. LOAD NPCs also exhibited reduced spare respiratory capacities both in untreated and NR+Caff treatment conditions, indicating diminished respiratory flexibility to stress, which could explain the lower activation of CAC, ETC, and MAS observed in these cells. NR+Caff treated LOAD astrocytes had similar levels of respiration and glycolysis as untreated astrocytes; however, all astrocytes exhibited an initial increase and subsequent decline of bioenergetic functions in the Biolog assay, which was consistent with an overall decrease in respiration or glycolysis in the Seahorse experiments 24 h post‐treatment. These data demonstrate that astrocytes, regardless of a LOAD background, have cell type‐specific bioenergetic reductions and long‐term reversal in response to NR and caffeine. There was a trend toward lesser bioenergetic functions in the NR+Caff treated LOAD astrocytes, which could be a consequence of reduced NAD redox factors in these cells. Fibroblasts behaved like astrocytes, showing decreases in respiration and glycolysis and biphasic metabolic profiles. However, there was a LOAD‐specific deficiency in response to NR and caffeine, as both young and old fibroblasts could increase bioenergetic substrate metabolism, while this was not the case for LOAD cells. Regardless, LOAD fibroblasts appear to have slightly better bioenergetic functions than old cells both in untreated and NR+Caff conditions, which could be a consequence of slightly increased mitochondrial mass, as previously reported (Sonntag et al., 2017). Importantly, in all LOAD cell populations, NR and caffeine treatment did not have a positive effect on cell viability or growth.

Together, these results demonstrate that in fibroblasts, NPCs, and astrocytes, NR and caffeine can temporarily boost NAD and bioenergetic functions, and elevated bioenergetic functions in LOAD astrocytes can be reduced to levels seen in untreated controls. However, NR and caffeine do not seem to have a substantial effect on improving longer‐lasting cellular energy management, or correct LOAD‐specific alterations in the production or transfer of reducing agents into the mitochondria and at the interphase of glycolysis and the mitochondrial respiratory chain, that is, deficiencies in activating CAC, ETC, G3P, and glycolysis, and increases in β‐oxidation, amino acid metabolism, and MAS (Ryu, Bormann, et al., 2021; Ryu, Cohen, et al., 2021). Instead, both Control and LOAD cells largely maintained their respective bioenergetic characteristics. The data from fibroblasts suggest that while there is still some remaining bioenergetic plasticity in old age, in LOAD there is substantially less responsiveness to NR and caffeine in activating the bioenergetic machinery, indicating disease‐specific deficiencies in maintaining bioenergetic plasticity. Altogether, supplementation with NR and caffeine had no substantial benefit on the bioenergetics or the viability or growth of cells with a LOAD background. In addition, the profound upregulation of NR or NAD‐degrading enzymes may cause unwanted adverse effects both at the cellular level as well as body‐wide when applied systemically. Our previous study suggested that the LOAD‐associated bioenergetic phenotype is inherent, that is in‐born, because we identified transcriptional changes in key factors related to altered bioenergetic functions in reprogrammed iPSC‐derived LOAD NPCs and astrocytes (Ryu, Bormann, et al., 2021). The observation that NR and/or caffeine do not influence these innate LOAD‐associated bioenergetic characteristics further supports a strong inherent, likely genetic, predisposition, difficult to modulate, in LOAD.

There are limitations to this study. Not all parameters and conditions can be concomitantly and tightly controlled in cell cultures. Many factors influence bioenergetic functions, such as glucose, lipid, and amino acid concentrations, oxygen tension, and others. In the Biolog assays, in which substrates are externally applied, the exact intracellular concentrations of individual metabolites cannot be determined. Saponin, however, used to permeabilize the cell membrane, had no apparent negative effect on cell survival. On the systems level, our cellular model does not recapitulate *in situ* brain physiology or pathophysiology and the brain's aging process. However, the iPSC‐derived NPCs and astrocytes are brain‐like cells and data from fibroblasts could indicate body‐wide alterations of cell function. As previously reported (Ryu, Cohen, et al., 2021), a cellular platform provides a translational tool to study LOAD pathogenic processes in context of age, disease, genetic background, cell development, and cell type. In addition, it can be used to investigate function‐modifying interventions, as demonstrated here for NR and caffeine, which could have implications for the design of future therapeutic strategies.

## 4 CONCLUSION

The results from our study demonstrate that despite increasing NAD redox factors and transiently enhancing some bioenergetic cell functions, in the end, NR and caffeine do not substantially improve energy management, in general, or address an innate LOAD bioenergetic phenotype. The consequences of NR and caffeine treatment on other cellular functions and neurodegeneration needs further investigation. However, our data suggest that targeting NAD alone may not be sufficient to improve energy metabolism in brain aging or ameliorate altered energy management associated with LOAD.

## 5 METHODS AND MATERIALS

### 5.1 Subject population, cell lines, and data usage

Subjects (Table S1) were recruited at the McLean Hospital Memory Diagnostic Clinic and diagnosed by a geriatric psychiatrist using the Diagnostic and Statistical Manual of Mental Disorders (DSM‐IV) criteria and the Montreal Cognitive Assessment (MOCA) score. Cell samples from LOAD patients and non‐demented control subjects were characterized and used in previous (McPhie et al., 2018; Ryu, Bormann, et al., 2021; Ryu, Cohen, et al., 2021; Sonntag et al., 2017; Yoshimizu et al., 2015) and the current study. Peripheral blood mononucleotide cells (PBMC) were isolated from blood samples. Fibroblasts were derived from skin biopsies as described (McPhie et al., 2018; Sonntag et al., 2017) and cultured in MEM media (Thermo Fisher Scientific) supplemented with 15% FBS, 100 U/ml Penicillin/Streptomycin (Thermo Fisher Scientific) and 2 mM GlutaMAX™ (Invitrogen). Cells were stored in the cell bank of the Program for Neuropsychiatric Research at McLean Hospital. Some data in untreated control conditions have been included in previous publications (Ryu, Bormann, et al., 2021; Ryu, Cohen, et al., 2021).

### 5.2 Generation of iPSC lines and culture

The generation and characterization of the iPSC lines were previously reported (McPhie et al., 2018; Ryu, Bormann, et al., 2021; Yoshimizu et al., 2015). Briefly, dermal fibroblasts or PBMCs were reprogrammed using the Sendai virus methodology, except for lines C1 and C2, which were converted by RNA reprogramming (McPhie et al., 2018; Yoshimizu et al., 2015). iPSC lines were expanded in feeder‐free cultures on Vitronectin (VTN; Gibco) coated plates in Essential 8™ Medium (Gibco) and propagated using ReLeSR (StemCell Technologies). All lines expressed stable normal karyotypes.

### 5.3 Generation of NPCs and astrocytes

Differentiation of iPSCs were as previously published (Ryu, Bormann, et al., 2021). iPSCs were dissociated to single cells and plated in AggreWell™ culture dishes (StemCell Technologies) to form embryonic bodies (EB) in Neural Induction Media (StemCell Technologies) supplemented with 2 μM dorsomorphin (Peprotech), 10 μM SB431542 (Peprotech), and 0.2 μM LDN193189 (Peprotech). At day in vitro (DIV) 5, EBs were transferred to VTN‐coated plates and cultured in the same media. Clusters of NPC‐containing rosettes were observed after DIV 6 and selectively picked and replated on VTN‐coated culture dishes. After growth to confluency, NPCs were plated at a density of 1–2 x 10^5^ cells/cm^2^ using Accutase™ (StemCell Technologies) and cultured in Neuro Basal Media (1:1 composition of Neurobasal A media (Gibco) and DMEM:F12 (Gibco)), supplemented with 1 × B27 supplement without Vitamin A (Gibco), 1× N2 supplement (Gibco), 1× NEAA (Invitrogen), 2 mM GlutaMax (Invitrogen), 1% Penicillin/Streptomycin (Invitrogen), 100 μM β ‐mercaptoethanol (Sigma‐Aldrich), 10 ng/ml EGF (Peprotech), and 10 ng/ml bFGF (Peprotech). NPCs were passaged every 5–7 days using Accutase and characterized for expression of SOX1, PAX6, and NESTIN by immunocytochemistry (ICC) at passage 3. To differentiate astrocytes, NPCs were plated at a density of 1 x 10^5^ cells/cm^2^ on VTN‐coated plates and, the next day, media was changed to astrocyte media (ScienCell) supplemented with 10 ng/ml BMP‐4, 10 ng/ml CNTF, and 10 ng/ml Heregulin‐β1 (all Peprotech) according to published protocols (Zhao et al., 2017). Astrocytes were grown to confluency and propagated every 5–7 days at a seeding density of 1 x 10^4^ cells/cm^2^ using Accutase. The cells were characterized for expression of GFAP, EEAT1, and S100β by ICC at DIV30.

### 5.4 Other cell lines

HFF1 cells were purchased from ATCC (HFF1 #SCRC‐1041) and secondary human astrocytes isolated from cerebral cortex were obtained from ScienCell Research Laboratories (#1800) (ScienCell). The cell lines were propagated in culture conditions recommended by the manufacturers.

### 5.5 NAD/NADH assay

NAD/NADH‐Glo™ Assay Kits (Promega) were performed on samples containing 2 x 10^5^ cells as previously published (Ryu, Bormann, et al., 2021; Ryu, Cohen, et al., 2021). Cells were lysed with 1% DTAB in 0.2 N NaOH, and half of the samples were prepared for measuring NAD^+^ by adding 0.4 N HCl (Sigma) and heating at 60°C for 15 min. After deactivating NADH in these samples, Trizma base (Sigma) was added to the acid‐treated samples. The other half of the samples was prepared to measure NADH by heating at 60°C for 15 min and adding HCl/Trizma solution. After the samples were prepared, luminescence was measured using a Synergy HT BioTek plate reader (BioTek). Data were normalized with protein measurement on lysed cells using the PierceTM BCA Protein Assay kit (Thermo Fisher). The NAD pool (total NAD) was calculated as follows: [NAD]total = [NAD^+^] + [NADH].

### 5.6 Seahorse assay

The Seahorse Mito Stress Tests determine oxygen consumption rate (OCR, pmol/min) and extracellular acidification rate (ECAR, mpH/min) after injection of specific pharmacologic stressors that target the electron transport chain (ETC) and ATP production: Oligomycin, which inhibits complex V (ATP synthase), decreasing OCR and ATP production; Carbonyl cyanite‐4 (trifluoromethoxy) phenylhydrazone (FCCP), which disrupts the mitochondrial membrane potential and collapses the proton gradient at the ETC leading to maximal respiration (O_2_ consumption by complex IV); and Rotenone/Antimycin A, which inhibit complex I and III resulting in the shutdown of mitochondrial respiration. From the data generated, several measures can be calculated, including basal and maximal respiration, and spare respiratory capacity (maximal respiration minus basal respiration), proton leak, non‐mitochondrial respiration, and the coupling effect, which determines ATP production relative to basal respiration. Extracellular acidification rate from which the proton efflux rate (PER, pmol H^+^/min) can be calculated are indirect measures of cell glycolytic capacity.

Seahorse XFp Cell Mito Stress Tests (Seahorse, Agilent Technologies) were performed on an XFp instrument as previously described (Ryu, Bormann, et al., 2021; Sonntag et al., 2017). Briefly, two days prior assay, Seahorse culture plates were coated with vitronectin for 1 h at 37°C and stored at 4°C. One day prior assaying, 20,000 NPCs and 10,000 fibroblasts or astrocytes were plated and cultured in the respective media overnight. On the day of the assay, XF assay medium was supplemented with 10 mM glucose, 1 mM pyruvate, and 2 mM glutamine, and the pH was adjusted to 7.4. After assay performance, cells were stained with CyQuant solution (Life Technologies ‐ Thermo Fisher Scientific) diluted in XF assay medium and incubated for 1 h at 37°C. Green fluorescence (excitation: 485/20, emission: 528/20) was measured using a Synergy HT BioTek plate reader (BioTek Instruments) and values used for data normalization. Data analysis was performed using the Seahorse XF^e^ Wave software, including the Seahorse XF Cell Energy Phenotype Test Report Generator. This algorithm determines the OCR and ECAR baseline phenotypes (measurement of the cells' relative utilization of mitochondrial respiration and glycolysis under starting conditions), the OCR and ECAR stressed phenotypes (measurement of the cells' relative utilization of mitochondrial respiration and glycolysis when stressed) and the respective metabolic potentials as percentage increase of stressed OCR or ECAR over baseline OCR or ECAR.

### 5.7 Biolog assay

Bioenergetics substrate metabolism was measured using Biolog MitoPlate S‐1 assays (Biolog) which assess mitochondrial function by measuring rates of electron flow into and through the ETC from 31 metabolic substrates that follow different pathways and transporters to enter the mitochondria and are processed by different dehydrogenases to produce NADH or FADH2. Electron transfer is monitored by a tetrazolium redox dye which acts as a terminal electron acceptor from cytochrome c. Metabolic substrates are processed in glycolysis, the PPP, directly or indirectly in CAC, G3P and MAS shuttles, β‐oxidation, or serve as amino acid carbon donors. Assays were performed according to manufacturer's instructions and as previously described (Ryu, Bormann, et al., 2021). MitoPlates were preincubated with 70 μg/ml saponin (Sigma, #47036), Biolog Mitochondrial assay solution, and Redox dye for 1 h at room temperature. After incubation, 60,000 NPCs and 40,000 fibroblasts or astrocytes per well were added to each plate and the OD 590 was measured at various times (0, 1, 2, 3, 4, 5, 6, 8, 16, 24, and 48 h) using a Synergy HT BioTek plate reader (BioTek). Measurements were normalized to and calculated as percent change from no substrate control, and the measured kinetic metabolic responses to NR+Caff were documented as percent change compared to untreated cells in Control and LOAD NPCs, astrocytes, and YC, OC, and LOAD fibroblasts.

### 5.8 Treatment with NR and caffeine

Prior to assaying, plated cells were cultured in their respective media for 2, 6, or 24 h in the presence or absence of NR (MedKoo Biosc. #329479) and caffeine (Sigma, #C0750), as described in the text.

### 5.9 qRT‐PCR

RNA extraction, cDNA preparation, and qRT‐PCR were performed as described (Ryu, Bormann, et al., 2021), using our published primers for NRK1, NMNAT2, and NAMPT (Ryu, Bormann, et al., 2021), and commercially available assays from BioRad for NAPRT1 (assay ID: qHasCID0020111), CD38 (assay ID: qHsaCED0041881), CD157 (BST1; assay ID: qHsaCED0056675), and SARM1 (assay ID: qHasCID0016061). Data were analyzed with the 2^−ΔCT^ method (Schmittgen & Livak, 2008).

### 5.10 Statistical analysis

Data were plotted as mean ± standard error of the mean (SEM) from at least 2 independent experiments performed in triplicates (*n* = 3), unless otherwise stated. One‐way analysis of variance (ANOVA) tests for independent measures were performed using Social Science Statistics software (http://www.socscistatistics.com/Default.aspx) or PRISM 8 for macOS Version 8.1.0. Differences were considered statistically significant when *p*‐values were less than 0.05, while *p*‐values between 0.05 and 0.1 were considered trend data.

## AUTHOR CONTRIBUTIONS

K.‐C.S. and B.M.C. conceptualized and designed, and B.M.C. funded the study. W.R., M.S., Y.L., R.A.H., and M.K.B., performed the experiments. W.R. and K.‐C.S. analyzed and interpreted the data, and K.‐C.S. wrote the paper. All authors read and approved the final manuscript.

## CONFLICT OF INTEREST

The authors declare no competing interests.

## CONSENT TO PARTICIPATE

Written informed consent provided by all subjects.

## CONSENT FOR PUBLICATION

All authors have given consent for publication.

## Supporting information


Appendix S1

## Data Availability

Data and material are made available upon request.
