# Supplementary material for: Nicotinamide riboside and caffeine partially restore diminished NAD availability but not altered energy metabolism in Alzheimer's disease
Source: Aging Cell. 2022 Jun 21;21(7):e13658. doi: 10.1111/acel.13658 (PMC9282847; doi:10.1111/acel.13658)
Supplement: Supplementary file 1 — Appendix S1 [file ACEL-21-e13658-s001.pdf]

Nicotinamide riboside and caffeine partially restore diminished NAD availability but not altered energy metabolism in Alzheimer's disease

Woo-In Ryu[1,2,3], Minqi Shen[1,2,3], Yoon Lee[1,2,3], Ryan A. Healy[1,2,3], Mariana K. Bormann[1,2,3], Bruce M. Cohen[1,3*], and Kai-C. Sonntag[1,2,3,4*]

[1]Department of Psychiatry, McLean Hospital, Harvard Medical School, Belmont, MA 02478, USA.
[2]Basic Neuroscience Division, McLean Hospital, Harvard Medical School, Belmont, MA 02478, USA.
[3]Program for Neuropsychiatric Research, McLean Hospital, Harvard Medical School, Belmont, MA 02478, USA.
[4]Contact author

*Correspondence to:
Kai-Christian Sonntag, M.D., Ph.D.,
Department of Psychiatry, Basic Neuroscience Division,
McLean Hospital, Harvard Medical School,
MRC 331, 115 Mill Street,
Belmont, MA 02478,
Phone: 617-855-3138,
Fax: 617-855-3793,
e.mail: ksonntag@mclean.harvard.edu

Bruce M. Cohen, M.D., Ph.D.,
Department of Psychiatry,
McLean Hospital, Harvard Medical School,
MRC 331, 115 Mill Street,
Belmont, MA 02478,
Phone: 617-855-2584
Fax: 617-855-3793,
e.mail: bcohen@mclean.harvard.edu

Supplementary Information

sample ID	sample ID iPSC	Age	Sex	Race	Diagnosis	APOE	Donor Cell iPSC	Karyotype	HIS*	MOCA*	MMSE*	Comorbidity
367		21	M	white	Ctrl	E3/3						No serious illness
368	C1	21	M	SE asian	Ctrl	E2/3	Dermal Fibroblast	normal, male				No serious illness
294		23	M	white	Ctrl	E2/2						No serious illness
460		25	M	white	Ctrl	E4/4						No serious illness
458		26	M	white	Ctrl	E2/3						No serious illness
359		31	M	black	Ctrl	E3/4						No serious illness
298		33	M	black	Ctrl	E2/3						No serious illness
366		35	M	white	Ctrl	E3/3						No serious illness
15	C2	37	M	white	Ctrl	E3/3	Dermal Fibroblast	normal, male				No serious illness
152		40	M	black	Ctrl	E3/3						No serious illness
190		42	M	white	Ctrl	E3/3						No serious illness
465		44	M	white	Ctrl	E3/3						No serious illness
226		45	M	white	Ctrl	E3/3						No serious illness
135		49	M	white	Ctrl	E3/3						No serious illness
365		52	M	white	Ctrl	E3/3						No serious illness
316		54	M	white	Ctrl	E3/3						No serious illness
14		55	M	white	Ctrl	E3/3						No serious illness
342	C3	57	M	white	Ctrl	E3/4	Dermal Fibroblast	normal, male				No serious illness
44	C6	59	M	white	Ctrl	E3/3	Dermal Fibroblast	normal, male				No serious illness, hypertension, cholesterol-reducing meds
498	C4	60	F	white	Ctrl	E3/3	Dermal Fibroblast	normal, female				No serious illness
491	C5	61	M	white	Ctrl	E3/4	Dermal Fibroblast	normal, male				No serious illness
497		61	F	white	Ctrl	E3/3						No serious illness
127		62	M	white	Ctrl	E3/4						Diabetes II, high BP, brain tumor
357		65	M	white	Ctrl	E3/3						No serious illness
47		65	F	white	Ctrl	E3/3						No serious illness
569		66	M	white	Ctrl	E3/3						No serious illness, rheumatoid arthritis on ibuprofen
205		67	M	white	Ctrl	E3/4						No serious illness, rheumatoid arthritis on ibuprofen
140	C7	72	M	white	Ctrl	E3/3	Dermal Fibroblast	normal, male				No serious illness
405	C8	75	F	white	Ctrl	E3/3	Dermal Fibroblast	normal, female				No serious illness, hypertension
169	C9	82	M	white	Ctrl	E3/3	Dermal Fibroblast	normal, male				No serious illness
563		88	M	white	Ctrl	E3/3						No serious illness
330	AD1	56	M	white	AD	E3/3	Dermal Fibroblast	normal, male	0	24		No serious illness
374		59	F	white	AD	E3/4			1	14		No serious illness
580		63	M	white	AD	E3/3			NA	NA	6	Diabetes II
572		64	F	white	AD	E3/4			2	6		No serious illness
231	AD2	65	M	white	AD	E3/4	Dermal Fibroblast	normal, male	NA	NA		Glaucoma, hypertension, CAD, cerebrovascular accident
402		70	F	white	AD	E3/4			2	18		Not recorded
399	AD3	71	F	white	AD	E4/4	Dermal Fibroblast	normal, female	2	10		No serious illness
BC2	AD7	71	M	white	AD	E3/4	PBMC	normal, male	0	NA	23	No serious illness
566		74	F	white	AD	E3/4			3	22		Hemochromatosis
369	AD4	76	M	white	AD	E3/4	Dermal Fibroblast	normal, male	1	13		Hemochromatosis
281	AD5	79	M	white	AD	E4/4	Dermal Fibroblast	normal, male	4	18		Diabetes II, hyperlipidemia, hypertension, CAD
404	AD6	81	M	white	AD	E3/4	Dermal Fibroblast	normal, male	1	5		No serious illness
BC3	AD8	78	F	white	AD	E3/4	PBMC	normal, female	2	NA	27	Diabetes II, hypertension, hyperlipidemia
332		82	F	white	AD	E3/3			1	14		No serious illness
BC4	AD9	>89	F	white	AD	E3/4	PBMC	normal, female	MRI	3		No serious illness

*HIS: Hachinsky Score
*MOCA: Montreal Cognitive Assessment Test
*MMSE: Mini-Mental State Exam

Table S1. List of cell lines and subject information.

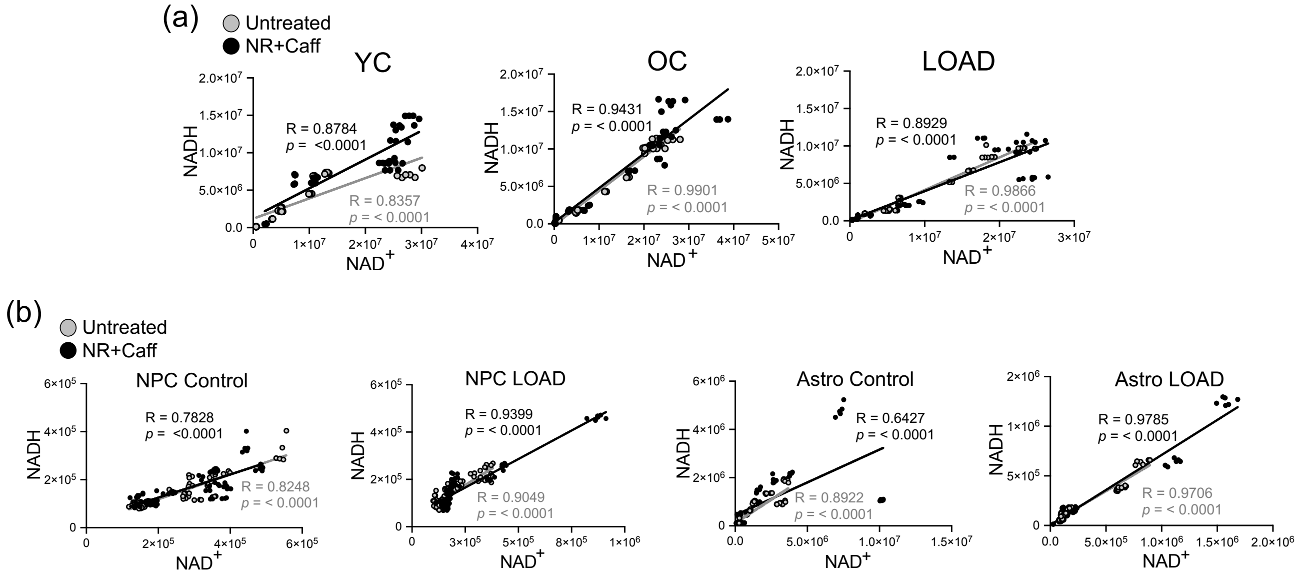

FIGURE S1 Pearson correlation coefficients for NAD^+ and NADH in untreated (gray) or NR+Caff treated (black) YC (*n* = 5, average age 25), OC (*n* = 8, average age 67), and LOAD (*n* = 8, average age 70) human skin fibroblasts (a), and in iPSC-derived Control (*n* = 9) and LOAD (*n* = 9) NPCs or astrocytes (b). R and *p* values are indicated.

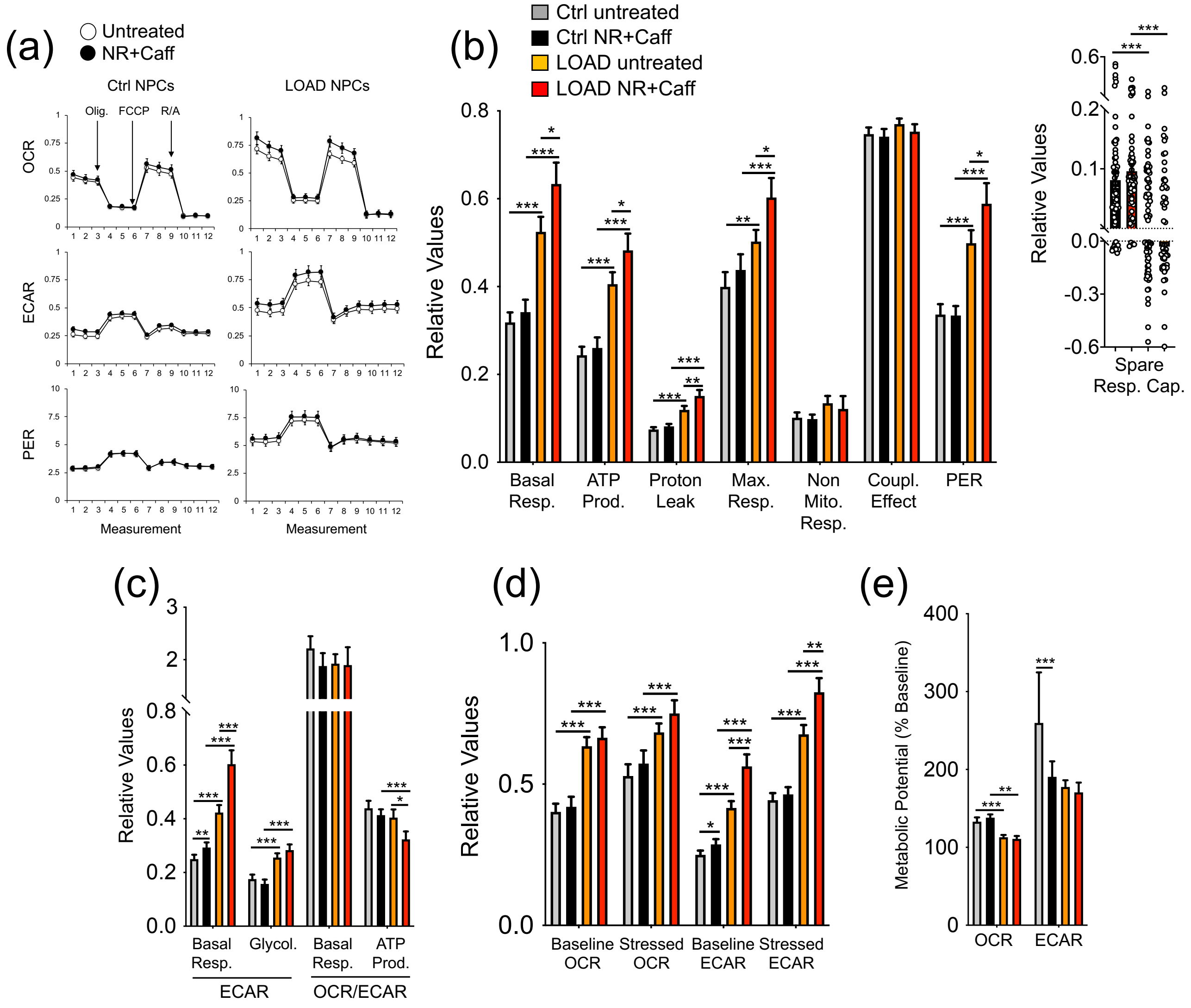

FIGURE S2 NR+Caff increases bioenergetic functions in iPSCs-derived NPCs**.** (a) Profiles of Seahorse XFp Mito Stress Test data for oxidative consumption rates (OCR, pmol/min), extracellular acidification rate (ECAR, mpH/min), and proton efflux rate (PER, pmol H+/min) in Control (*n* = 9) and LOAD (*n* = 9) NPCs in untreated (white) condition or after treatment with NR+Caff (black) for 24 hours. Arrows indicate injections of specific stressors of mitochondrial respiration, including oligomycin (Olig.), carbonyl cyanite-4 (trifluoromethoxy) phenylhydrazone (FCCP), and Rotenone/Antimycin A (R/A). (b, c) Relative values of OCR (pmol/min) (b), ECAR (mpH/min) and OCR/ECAR (c) for Control and LOAD NPCs in untreated and NR+Caff treatment conditions. (d, e) Results from Cell Energy Phenotype Test Report Generator for Control and LOAD NPCs plotted as relative values baseline and stressed OCR or ECAR (d), and their calculated metabolic potentials ((stressed OCR or ECAR / baseline OCR or ECAR) x 100%)) (e).

Data are means +/- SEM from two repeat experiments. *$p < 0.1$; **$p < 0.05$; ***$p < 0.01$ using one-way ANOVA, depicting significant changes between treated and untreated cells or groups.

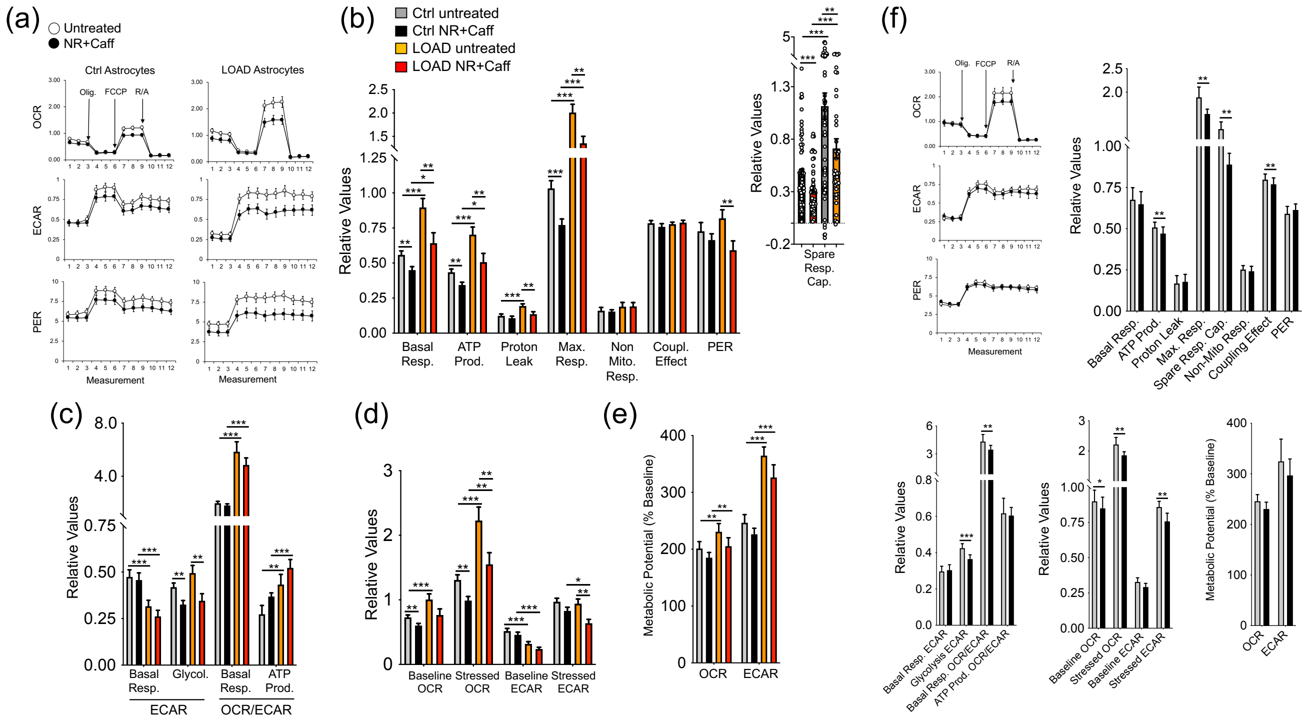

FIGURE S3 NR+Caff decreases bioenergetic functions in iPSC-derived astrocytes. (a) Profiles of Seahorse XFp Mito Stress Test data for oxidative consumption rates (OCR, pmol/min), extracellular acidification rate (ECAR, mpH/min), and proton efflux rate (PER, pmol H+/min) in Control (*n* = 9) and LOAD (*n* = 9) astrocytes in untreated (white) condition or after treatment with NR+Caff (black) for 24 hours. Arrows indicate injections of specific stressors of mitochondrial respiration, including oligomycin (Olig.), carbonyl cyanite-4 (trifluoromethoxy) phenylhydrazone (FCCP), and Rotenone/Antimycin A (R/A). (b, c) Relative values of OCR (pmol/min) (b), ECAR (mpH/min) and OCR/ECAR (c) for Control and LOAD NPCs in untreated and NR+Caff treatment conditions. (d - e) Results from Cell Energy Phenotype Test Report Generator for Control and LOAD NPCs plotted as relative values baseline and stressed OCR or ECAR (d), and their calculated metabolic potentials ((stressed OCR or ECAR / baseline OCR or ECAR) x 100%)) (e). (f) Profiles of Seahorse XFp Mito Stress Test for secondary human astrocytes.

Data are means +/- SEM from two repeat experiments. **p* < 0.1; ***p* < 0.05; ****p* < 0.01 using one-way ANOVA, depicting significant changes between treated and untreated cells or groups.

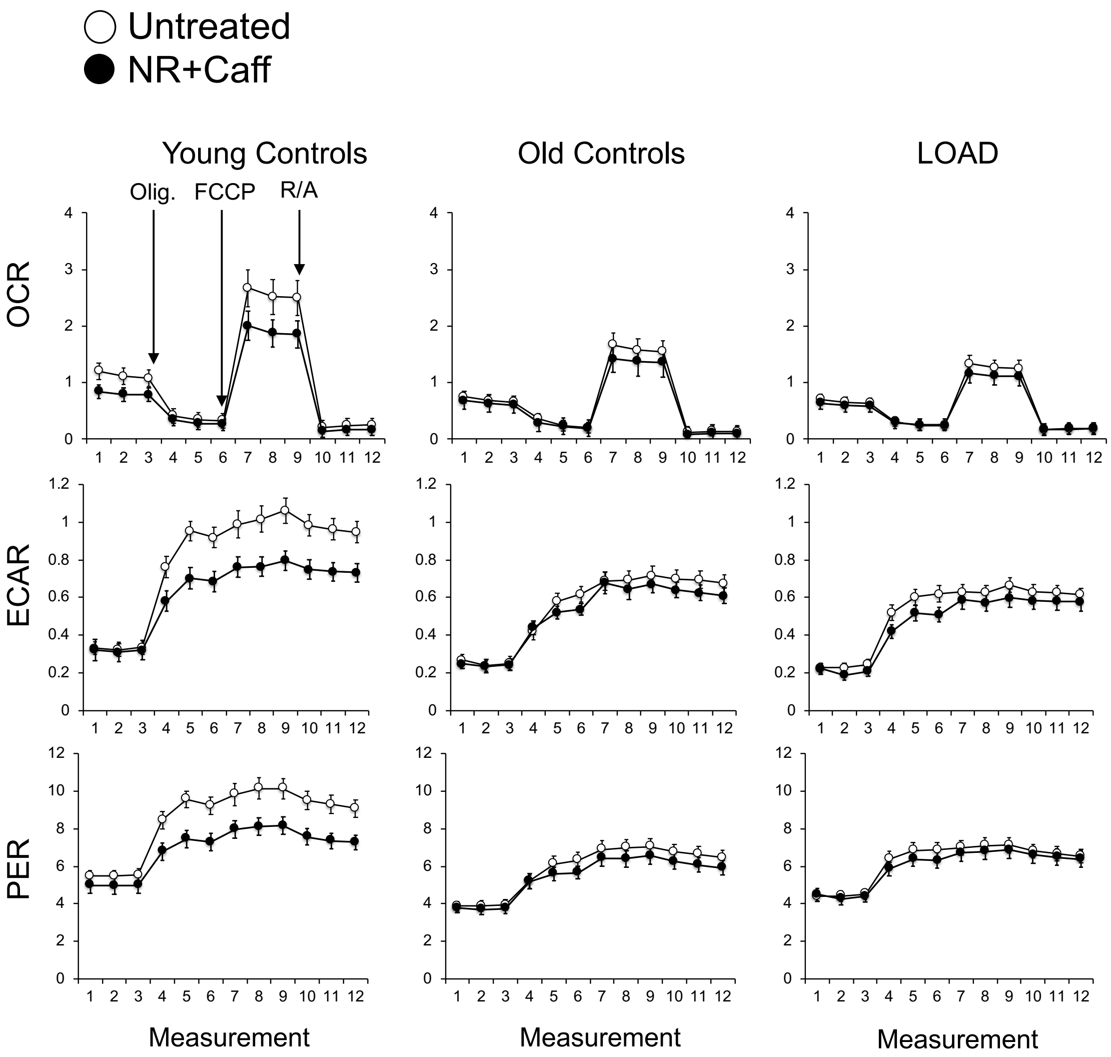

FIGURE S4 NR+Caff decreases bioenergetic functions in skin fibroblasts. (a) Profiles of Seahorse XFp Mito Stress Test data for oxidative consumption rates (OCR, pmol/min), extracellular acidification rate (ECAR, mpH/min), and proton efflux rate (PER, pmol H+/min) in YC (*n* = 17, average age 37), OC (*n* = 11, average age 66), and LOAD (*n* = 10, average age 71) in untreated (white) condition or after treatment with NR+Caff (black) for 24 hours. Arrows indicate injections of specific stressors of mitochondrial respiration, including oligomycin (Olig.), carbonyl cyanite-4 (trifluoromethoxy) phenylhydrazone (FCCP), and Rotenone/Antimycin A (R/A).

Data are means +/- SEM from two repeat experiments. *$p < 0.1$; **$p < 0.05$; ***$p < 0.01$ using one-way ANOVA.

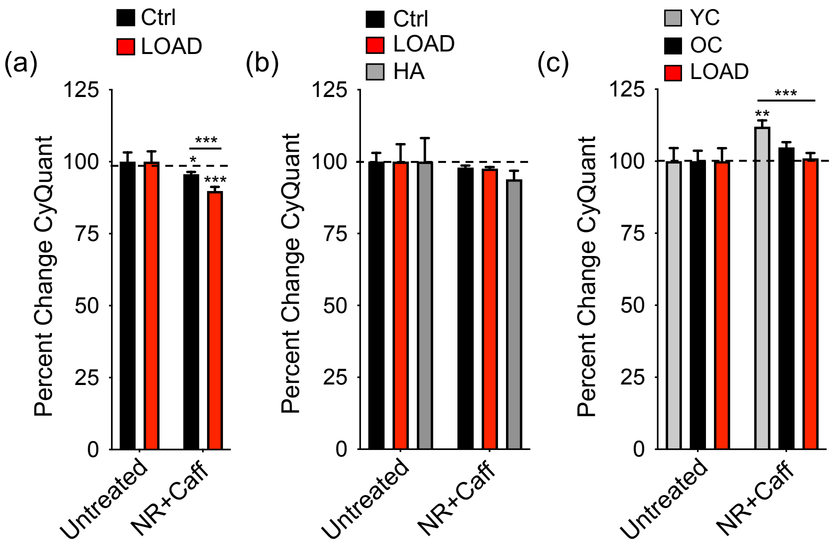

FIGURE S5 NR+Caff has different effects on cell growth of NPCs, astrocytes, and fibroblasts. (a - c) CyQuant measurements plotted as percent change fluorescence in NR+Caff over untreated Control (black) and LOAD (red) NPCs (a), Control (black), LOAD (red), and secondary human (HA) (gray) astrocytes (b), and YC (gray), OC (black), and LOAD (red) fibroblasts (c).
Data are means +/- SEM from two repeat experiments in iPSC-derived Control and LOAD NPCs and astrocytes (n = 9 samples in each group), and n = 17 YC (average age 37), n = 11 OC (average age 66), and n = 10 LOAD (average age 71) fibroblast lines, and from four repeat experiments in HA. *$p < 0.1$; **$p < 0.05$; ***$p < 0.01$ using one-way ANOVA, depicting significant changes between treated and untreated cells or groups.

REFERENCES

Sonntag, K.-C., Ryu, W.-I., Amirault, K. M., Healy, R. A., Siegel, A. J., McPhie, D. L., . . . Cohen, B. M. (2017). Late-onset Alzheimer's disease is associated with inherent changes in bioenergetics profiles. *Scientific reports, 7*(1), 1-13.
